# Use of external quality assessment in extra-analytical phases in clinical laboratories in Spain: a survey by the Spanish Society of Laboratory Medicine (SEQC^ML^)

**DOI:** 10.1515/almed-2025-0067

**Published:** 2025-04-21

**Authors:** Andrea Caballero Garralda, Immaculada Comas Reixach, Carlos García Miralles, Rubén Gómez Rioja, María Antonia Llopis Díaz, Débora Martínez Espartosa, Reyes Nicolás de Blas, Mariona Panadès Turró, Laura Puigví Fernández, Laura Rodelgo Jiménez, Berta Sufrate-Vergara, Emma Ventura Orriols

**Affiliations:** Extraanalytical Quality Commission of the Spanish Society of Laboratory Medicine (SEQC^ML^), Department of Clinical Biochemistry, Vall d’Hebron Hospital, Barcelona, Spain; Extraanalytical Quality Commission of the Spanish Society of Laboratory Medicine (SEQC^ML^), Department of Clinical Biochemistry, Parc Tauli Hospital Universitari, Sabadell, Spain; Extraanalytical Quality Commission of the Spanish Society of Laboratory Medicine (SEQC^ML^), Department of Laboratory Medicine, La Paz-Cantoblanco-Carlos III Hospital, Madrid, Spain; Extraanalytical Quality Commission of the Spanish Society of Laboratory Medicine (SEQC^ML^), Catalonian Institute of Health: Barcelona, Spain; Extraanalytical Quality Commission of the Spanish Society of Laboratory Medicine (SEQC^ML^), Department of Clinical Biochemistry, Clínica Universidad de Navarra, Madrid, Spain; Extraanalytical Quality Commission of the Spanish Society of Laboratory Medicine (SEQC^ML^), Department of Clinical Biochemistry, Ramón y Cajal University Hospital, Madrid, Spain; Extraanalytical Quality Commission of the Spanish Society of Laboratory Medicine (SEQC^ML^), Quality Manager-Spanish Society of Laboratory Medicine (SEQC^ML^), Barcelona, Spain; Extraanalytical Quality Commission of the Spanish Society of Laboratory Medicine (SEQCML), Extraanalytical Head Department, Director of Operations, CLILAB Diagnòstics, Barcelona, Spain; Extraanalytical Quality Commission of the Spanish Society of Laboratory Medicine (SEQC^ML^), Institut of Laboratory Medicine, San Carlos Clinic Hospital, Madrid, Spain; Extraanalytical Quality Commission of the Spanish Society of Laboratory Medicine (SEQC^ML^), Extraanalytical Department of Laboratory, Hospital Sant Joan de Deu, Esplugues de Llobregat, Barcelona, Spain

**Keywords:** extra-analytical phase, quality indicators, external quality assurance program, survey

## Abstract

**Objectives:**

Quality indicators (QIs) are a valuable tool for monitoring and improving extra-analytical phase performance in the clinical laboratory (CL). For that reason, the Spanish Society of Laboratory Medicine (SEQC^ML^) launched its Pre-analytical Phase EQA Program in 2001.

**Methods:**

In 2023, a survey was conducted to assess the level of participation in the EQA Program, identify its potential improvements, and enhance the understanding of the collection and estimation processes of the extra-analytical QI.

**Results:**

In total, 51 % of respondents (124 responses in total) were enrolled in the program. The primary reason for participating was the need of monitoring of the extra-analytical phases (38 %). In contrast, the main reason for not taking part in the EQA Program was the difficulty in collecting data (32 %). As to potential points for improvement, 56 % considered that QI should be analyzed disaggregated by type of extraction (ambulatory vs. urgent). As many as 86 % of respondents would be interested in participating in a post-analytical phase EQA Program. Most of the suggestions about indicators to be included were related to events regarding temperature and transport times from peripheral extraction sites.

**Conclusions:**

Although interest in QIs for pre- and post-analytical phases is growing, the percentage of laboratories that collect data regularly is still limited. The findings of this survey have led the future directions for the EQA Pre-analytical Program, updating and adapting it to the current needs of CLs.

## Introduction

Most errors in the clinical laboratory (CL) occur in the pre- and post-analytical phases of the total testing process (TTP), which jeopardizes patient safety [1]. Although the concept of brain-to-brain loop was first described over 43 years ago [2], it has not been until recently that awareness and consensus about the impact of extra-analytical phases on laboratory results became widespread [3], 4]. Continuous monitoring and assessment of the entire TTP are essential for quality laboratory results to be obtained [5].

The implementation of an internal quality monitoring system [6] and participation in external quality assurance (EQA) programs are crucial for continuous improvement [7]. The integration of quality indicators (QI) in ongoing improvement schemes has been recognized to be a useful tool for monitoring and improving extra-analytical performance [8], 9]. According to the ISO 15189:2023 standard, the identification and use of QI throughout the TTP is an essential requirement for accreditation [10], including the use of QI to assess performance in pre- and post-analytical phases [11].

For that reason, in 2001, the Spanish Society of Laboratory Medicine (SEQC^ML^) launched the Pre-Analytical EQA Program, which initially consisted of cross-sectional studies [12], 13]. From 2014, this Program was transformed into a Pre-analytical QI Benchmarking Program. The Program explores a reduced number of QI based on the most prevalent events detected in the previous phase of the Program. Special focus is placed on the reasons for sample rejection at the laboratory. In this Program, only routine test results are considered, whereas urgent test results are excluded. The denominator used is the most common type of test used for each type of specimen (i.e. creatinine for serum; CBC for EDTA; and prothrombin time for citrate; the total number of tubes is only used for urine). These QI are specially focused on the sample extraction (venous puncture) and collection process. These processes can be improved by enhancing the training of personnel involved in the proper monitoring of processes.

The purpose of this Program is to improve pre-analytical phases by reducing errors with a potential impact on patient safety. In general terms, the same QI have been used for years, which has facilitated the development of specifications based on the state-of-the-art, as suggested in the European Federation of Laboratory Medicine (EFLM) recommendations (Task Finish Group – Performance specifications for the extra-analytical phases). According to these recommendations, percentiles p25, p50 and p75 from participant results are adopted as optimal, desirable and minimum specifications, respectively.

On another note, CLs are striving for laboratory results to have the greatest possible impact on patient outcomes. Most recently, CLs have placed the focus on the post-analytical phase and how the laboratory can add value to laboratory results (interpretative comments, critical result alerts, turnaround times, among others) [14], 15]. In this sense, the Working Group for Post-analytics of the Croatian Society of Medical Biochemistry and Laboratory Medicine conducted a survey to assess post-analytical practices and evaluate national recommendations [16].

In addition, the advent of new technologies, laboratory information systems (LIS), and increasing process automation makes it necessary to review the validity of the QI currently used for monitoring laboratory performance. The EFLM Working Group for the Pre-analytical Phase conducted a survey to assess European laboratory practices in monitoring the pre-analytical phase [17], 18]. The survey revealed that 71 % of laboratories were enrolled in an extra-analytical EQA program. However, a very low number of the laboratories engaged in a SEQC^ML^ Quality Assurance Program (PGCLC) participated in the Extra-Analytical Quality Assurance Program.

For this reason, a survey was conducted among CLs taking part in any PGCLC scheme. The aim of the Survey was to assess the level of participation in the Program and identify potential points for improvement. Other objectives included assessing the procedures used and difficulties faced by laboratories in relation to the collection and estimation of extra-analytical QI. Finally, the Survey examined whether the QI currently used meet the needs of increasingly automated and complex CL.

## Materials and methods

### Survey design

The Survey was designed by the members of the SEQC^ML^ Extra-analytical Quality Commission. It consisted of 48 dichotomous/multiple-choice/open questions and was developed on the Google forms platform. The Survey was distributed as a pilot to the members of the Extra-analytical Quality Commission, whose contributions were used to refine its design. All questions and answer options are shown in Table 1 of the Supplementary Material.

**Table 1:** Summary of the main results of the survey.

Annual requests	
**Responses (n=124)**	**%**
<25,000	17
25,000–300,000	41
>300,000	42

**Types of requests**	

**Responses (**n=**124)**	**%**
Routine ambulatory	31
Ambulatory ED	17
Routine hospital	26
Urgent hospital	26

**Percentage of external samples**	

**Responses (**n=**124)**	**%**
<33 %	26
33–66 %	43
>66 %	31

**Review of indicators disaggregated by type of extraction**	

**Responses (**n=**124)**	**%**
Yes	56
No	44

**Interest in including sigma**	

**Responses (**n=**120)** ^**a**^	**%**
Yes	74
No	26

**Interest in the post-analytical phase**	

**Responses (**n=**124)**	**%**
Yes	86
No	14

**Interest in including ED laboratory samples**	

**Responses** ^**b**^	**%**
Yes, combining urgent and routine requests	37
Yes, separating urgent and routine requests	44
No	19

^a^Of laboratories taking part in other programs, 1 yes has sigma and 3 no. ^b^The four remaining responses are from laboratories engaged in other programs. All received urgent requests.

### Survey distribution

The Survey was distributed to PGCLC-participating laboratories (n=2,409) via e-mail and a post on LinkedIn, both including a link to complete the questionnaire on *Google forms*. The questionnaire was left open for two months and a half, from June 19, 2023 to August 31, 2023.

### Survey analysis

Graphs were created to analyze the results of close-ended questions. Responses to open questions were analyzed one by one, grouped by themes.

## Results

A total of 124 questionnaires were returned, accounting to a 5 % response rate, which is consistent with the response rate of previous surveys conducted by the Commission [19]. Table 1 summarizes the most relevant findings.

In relation to the characteristics of respondents, 42 % were members of large laboratories receiving over 300,000 requests on an annual basis; 41 % belonged to medium laboratories (25,000–300,000), and the remaining worked at small laboratories receiving less than 25,000 annual requests. Laboratories reported that they receive a similar number of routine and urgent outpatient and inpatient test orders. In most laboratories (43 %), 33–66 % of samples were external (samples collected at a center other than the laboratory’s).

**Figure 1:**
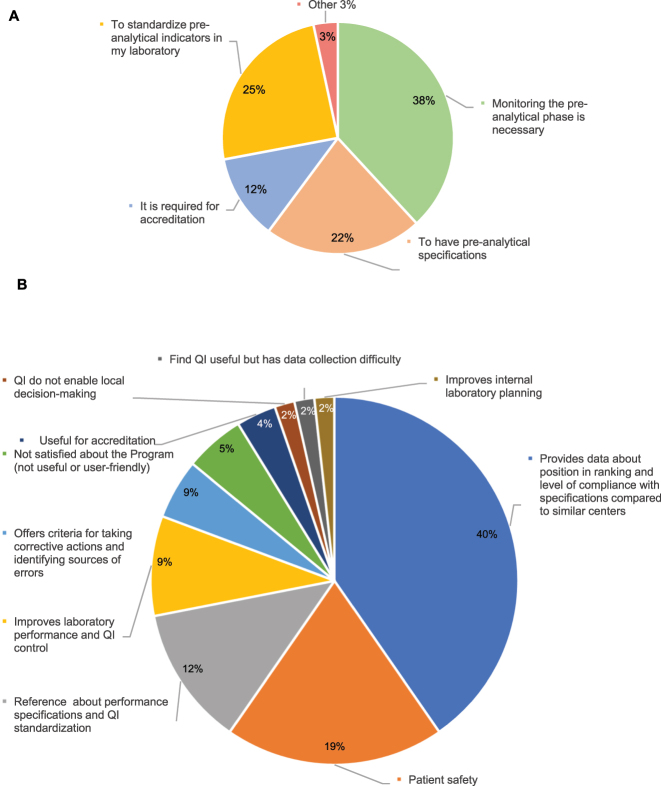
Results for the participating laboratories in the pre-analytical program (63 laboratories). (A) Reasons for taking part. (B) Main contributions of the program.

**Figure 2:**
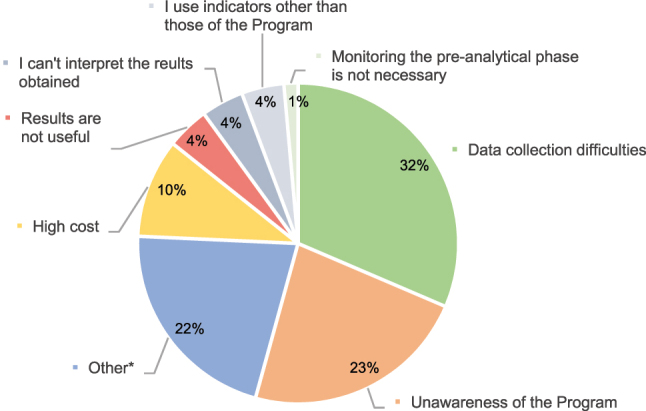
Reasons for laboratories not participating in the pre-analytical program (57 laboratories). *Other: (1) indices are not available on the LIS. (2) The laboratory company does not participate. (3) We would like our customers to participate. (4) No specific reason. (5) I took part some time ago but did not agree with the reported results. (6) We only take part in the fecal occult blood program. (7) I work at the ED laboratory. (8) Lack of time. (9) Only internal pre-analytical quality monitoring is performed. (10) We did not receive any offer from the organizing entity. (11) The performance specifications used. (12) Serum indices are not available in the laboratory and an automated request and report system is not available.

Regarding the participation in the EQA Program, 51 % percent of respondents were engaged in the Pre-analytical EQA Program, 46 % did not participate in it, and 3 % were engaged in EQA Programs offered by other entities. The main reason to participate in the Programs was that they considered it necessary to monitor extra-analytical performance (38 %), harmonize QIs (25 %), and establish performance specifications (22 %). The least frequent response was the need to participate in the Program due to accreditation requirements. In total, 40 % of participants considered that the main advantage of participating in the program was that it provides information about the position of the laboratory in the ranking and provides an insight into the level of compliance of specifications with respect to similar laboratories (40 %), followed by patient safety (19 %) (Figure 1). In contrast, the main reason stated by non-participating laboratories (57) for not being engaged in the Program was the difficulty in collecting data (32 %), followed by unawareness of the availability of this Program (23 %) (Figure 2).

**Figure 3:**
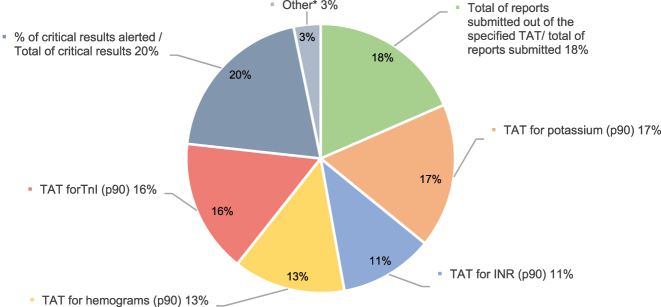
Interest in post-analytical indicators. TAT: turnaround time from receipt at the laboratory to the communication of results for urgent samples. *Other: (1) indicators related to consultations from clinicians to the laboratory and with result alerts; (2) routine inpatient and outpatient samples; (3) percentage of reports modified/total of reports; (4) critical rejections timely communicated/total of critical rejections detected; (5) number of poorly sent-validated reports/total reports (6) turnaround time from receipt at the laboratory to the communication of results for venous and arterial blood gas tests; (7) indicators related to the communication of results for procalcitonin in urgent samples; (8) interpretative results/diagnostic opinion (% of lack of correlation between conclusive results and % of correlation between clinical picture and the tests performed); (9) indicators related to the transcription of results (% of errors in result transcription and % of untimely transcription of results).

In relation to potential improvements to the Program, 56 % suggested that disaggregation of QI by type of extraction (inpatient vs. outpatient) would be useful. More specifically, most participants supported the inclusion of urgent test requests in QI assessment, separated from routine test requests. Conversely, 37 % of respondents would combine them with routine test, and 19 % would exclude them. Urgent samples are considered in all extra-analytical EQA Programs other than the SEQC^ML^ Program. In total, 74 % would include Sigma estimates. Notably, Sigma estimates are included in only one of the four non-SEQCML programs available. Interestingly, 86 % of respondents showed interest in participating in a post-analytical quality assessment program, one of the open-ended questions surveyed which post-analytical QIs (Figure 3). The most selected indicators included the percentage of critical alerts raised with respect to the total critical result alerts and the number of reports delivered out of the turnaround time. Other indicators frequently selected by respondents were related to report modifications, interpretative comments, result transcription and the delivery of reports containing errors.

The second part of the Survey was focused on QI data collection and estimation by laboratories. In 73 % of cases, this process is performed via the LIS, followed by manual counting (on an Excel spreadsheet or similar) (19 %), with the rest of laboratories using dedicated software. LIS suppliers included Werfen Modulab^®^ (23 %), Roche Infinity^®^ (19 %) and Siemens Healthineers Servolab^®^ (14 %), with other suppliers accounting for less than 10 %.

Finally, interest was shown for some QI not assessed in the Program, related to events regarding temperatures and transport times from peripheral extraction centers. Also, suggestions were made about applying some QI already included in the Program to other types of samples (blood gas test syringes, urine for urine analysis, other microbiology samples, among others). Respondents showed interest in QI related to sample transport, since 33–66 % of samples received by laboratories are external. The management of such a high percentage of external samples requires close coordination with external centers, the inclusion of ED laboratory data and clearer instructions for QI collection, along with a modified denominator for calculating QI, and the inclusion of other types of samples.

## Discussion

Participation in external quality assurance programs for the pre-analytical phase facilitates the identification and prioritization of areas for improvement. The participants, most being large laboratories receiving over 300,000 requests a day, indicated that the SEQC^ML^ External Quality Assurance Program for the Pre-Analytical Phase is a valuable tool.

The monitoring of pre-analytical errors through robust QI is essential for their proper management. Additionally, external benchmarking enables laboratories to assess their performance against standard specifications. As a result, the number of errors decreases, thereby improving patient safety.

The results of this Survey demonstrate that participation of clinical laboratories in Extra-Analytical Programs is still low. Only half of clinical laboratories in Spain participate in these Programs, compared to 71 % in Europe, as shown in a similar Survey (17). The main reason for not taking part in these programs is the difficulty in IQ data collection, followed by unawareness of the availability of these Programs. Another possible reason for such a low level of participation in Extra-Analytical Quality Programs is the reluctance to disclose organizational data, which may negatively affect their image or relationship with customers. Although confidentiality is ensured in this Program, a negative bias in participation may arise for this reason.

Based on the results of this Survey, a campaign was launched on the PGCLC website and SEQC^ML^ social networks. The results of the Survey highlight the need for cooperating with the leading LIS suppliers to create modules for a simpler, standardized, automated, real-time calculation of QI that can be easily shared and assessed. The Survey revealed that 19 % of laboratories still rely on manual methods for QI calculation, which poses the opportunity to implement more automated LIC-based solutions.

Based on the interest shown by participants in including urgent samples along with post-analytical and sample transport QI, the SEQC^ML^ Extra-analytical Quality Commission and the External Quality Programs Committee conducted a pilot study among its members. Following the obtained results, they both decided to further include urgent samples for all Program participants over 2025. For such purpose, the number of QI doubled. Participants will receive two reports, one for routine samples and another for urgent samples. Separated reports will allow participating laboratories to compare the two processes and establish targeted corrective measures.

In a second phase, two more QI will be integrated in the Program, in response of the answers obtained, such as the generalized interest in the post-analytical phase and the addition of more pre-analytical QI different from the ones currently used. The two new QI include the compliance with the turnaround time and critical result alerts. Furthermore, another pre-analytical QI about transport events from peripheral extraction samples will be included, following the same scheme as the pilot study with a progressive implementation over 2026.

With respect to other programs, the *Working Group Laboratory Errors and Patient Safety* (WG-LEPS) of the *International Federation of Clinical Chemistry* (IFCC) has not released any further publication since 2019 [20] nor has the *Australian key incident monitoring and management system program* (KIMMS) of the *Royal College of Australasian Pathologist* (RCPA) [21] since 2020. The results of these programs were already compared against the SEQC^ML^ Preanalytical Program in a publication of 2022 [22]. Notably, two recent experiences deserve special attention, one of the Argentine Foundation for Biochemistry [23] and another in China [24]. The first one was a quarterly survey where only outpatient samples and four QI were included. It was conducted in the framework of a larger program and indicators were entered onto an Excel spreadsheet. Participation was voluntary, resulting in a varying percentage of laboratories, with differences according to the QI. Results are provided for the 2021–2022 period. Between 100 and 200 out of a total of 400 laboratories returned the questionnaire (25–50 %), with similar results if percentiles are compared against those of the IFCC (23). In the Survey of the *National Centre for Clinical Laboratories of China*, two questionnaires assessing six QI were distributed annually via email over the 2017–2019 period. In total, 434 laboratories of the province of Zhejiang responded to the six runs. The rate of rejection was very low, as compared to the Q-Probes and Q-Tracks Program of the College of American Pathologist (CAP).

A significant limitation of the Survey conducted with SEQCML laboratories is the low participation. Although this is not a particularity of this Survey and the reasons cannot be clearly identified (overexposure to surveys? Limited time of laboratory staff? Low interest in this issue?), a change of format should be considered for future surveys. Other strategies to be adopted include increasing survey dissemination and improving access through different channels to enhance response rates and the power of the studies.

There is a growing interest in QIs related to the pre- and post-analytical phases. However, a reduced proportion of laboratories collect data regularly. The findings of this Survey suggest future directions for new developments and optimization of the SEQC^ML^ Pre-analytical EQA Program to better adapt to the current needs of clinical laboratories. Data collection difficulty is the main reason for such a low level of participation. Hence, further collaboration with LIS suppliers is necessary for the development of new, simpler data collection tools. Furthermore, it is the responsibility of laboratories to allocate more resources, personnel and funds to the extra-analytical phase.

In conclusion, the monitoring of pre-analytical errors through robust QI is essential for their proper management. Additionally, external benchmarking enables laboratories to assess their performance against standard specifications. As a result, the number of errors decrease and patient safety is improved.

## Supplementary Material

Supplementary Material
