# Supplementary material for: Use of external quality assessment in extra-analytical phases in clinical laboratories in Spain: a survey by the Spanish Society of Laboratory Medicine (SEQCML)
Source: Adv Lab Med. 2025 Apr 21;6(2):199–205. doi: 10.1515/almed-2025-0067 (PMC12107415; doi:10.1515/almed-2025-0067)
Supplement: Supplementary file 1 — Supplementary Material [file j_almed-2025-0067_suppl_001.doc]

**SUPPLEMENTARY MATERIAL**

**Table 1.** Survey Questions**.**

**Do you participate in the SEQC-ML External Pre-analytical Quality Assurance Program?**

1. Yes
2. No, I participate in the External Pre-analytical Quality Assurance Program of another entity.
3. No, I do not participate in any External Pre-analytical Quality Assurance Program.

**Yes, I participate in the SEQC-ML External Pre-analytical Quality Assurance Program**

**What is your main reason for participating?**

1. Monitoring the pre-analytical phase is necessary
2. To have pre-analytical performance specifications available
3. It is required for accreditation
4. To standardize preanalytical quality indicators in my laboratory
5. Other

**If your laboratory collects pre-analytical indicators other than those in the Program, please, indicate which ones:**

**How do you collect the data required for calculating the indicators?**

1. Through the Laboratory Information System
2. By manual counting in Excel (or similar)
3. By retrieving data from the Information System of the centre
4. Other

**What is your LIS supplier?**

**When you receive the results, does your laboratory review the indicators disaggregated by type of extraction centre? For example, if extractions are performed at different primary care centres and an indicator is elevated, do you review the result obtained in each centre for that indicator?**

1. Yes
2. No

**What does the Pre-analytical Program offer?**

**What aspects of interest would you add or modify?**

**Would it be useful to include Sigma calculations in the Program?**

1. Yes
2. No

**Would it be useful that the Program was extended to urgent samples?**

1. Yes, it would be useful to include indicators for urgent samples along with routine samples in the same Program
2. Yes, a Pre-analytical program that included urgent samples separately from routine samples would be useful
3. No

**Would you be interested in participating in a Post-analytical Program?**

1. Yes
2. No

**What post-analytical indicators would be more useful for you?**

1. Number of reports returned out of the specified deadline/Total of reports returned
2. Turnaround time from receipt at the laboratory to the communication of results for potassium in urgent samples (p90)
3. Turnaround time from receipt at the laboratory to the communication of results for INR in urgent samples (p90)
4. Turnaround time from receipt at the laboratory to the communication of results for the hemogram in urgent samples (p90)
5. Turnaround time from receipt at the laboratory to the communication of results for TnI in urgent samples (p90)
6. Percentage of critical result alerts/Total of critical alerts raised
7. Other

**In relation to your laboratory, please mark the number of requests received on an annual basis**

1. < 25,000
2. 25,000 - 300,000
3. 300,000

**In relation to your laboratory, please mark the type of requests received**

1. Routine outpatient
2. Urgent outpatient
3. Routine inpatient
4. Urgent inpatient

**What percentage of external samples does your laboratory receive? External samples are defined as samples not extracted at the centre.**

1. < 33%
2. 33 - 66%
3. 66%

**Name of the laboratory**

**Comments:**

**It participates in the External Pre-analytical Quality Assurance Program of another entity.**

**In which program do you participate?**

**What does this Program offer compared to the SEQC-ML Preanalytical Program?**

**What aspects of interest would you add to or modify in your Program?**

**What is the main reason for participating in a Pre-analytical Program?**

1. Monitoring the pre-analytical phase is necessary
2. To have pre-analytical performance specifications
3. It is required for certification
4. To standardize preanalytical indicators in my laboratory
5. Other

**If your laboratory collects pre-analytical QI other than those in the Program, please, indicate which ones:**

**How do you collect the data required for calculating the indicators?**

1. Through the Laboratory Information System
2. By manual counting on Excel (or similar)
3. By retrieving data from the Information System of the centre
4. Other

**What is your LIS supplier?**

**When you receive the results, does your laboratory review the indicators disaggregated by type of extraction centre? For example, if extractions are performed at different primary care centres and an indicator is elevated, do you review the result obtained for that indicator at each centre?**

1. Yes
2. No

**Does your Program include Sigma calculation?**

1. Yes
2. No

**Does your Program assess urgent samples?**

1. Yes
2. No

**Would you be interested in participating in a Post-analytical Program?**

1. Yes
2. No

**What post-analytical indicators would be more useful for you?**

1. Number of reports returned out of the specified deadline/Total of reports returned
2. Turnaround time from receipt at the laboratory to the reporting of results for potassium in urgent samples (p90)
3. Turnaround time from receipt at the laboratory to the reporting of results for INR in urgent samples (p90)
4. Turnaround time from receipt at the laboratory to the reporting of results for the hemogram in urgent samples (p90)
5. Turnaround time from receipt at the laboratory to the communication of results for TnI in urgent samples (p90)
6. Percentage of critical results alerted/Total of critical alerts raised
7. Other

**In relation to your laboratory, please mark the number of requests received annually**

1. < 25,000
2. 25,000 - 300,000
3. 300,000

**In relation to your laboratory, please mark the type of requests received**

1. Routine outpatient
2. Urgent outpatient
3. Routine inpatient
4. Urgent inpatient

**What percentage of external samples does your laboratory receive? External samples are defined as samples not extracted at the centre.**

1. < 33%
2. 33 - 66%
3. 66%

**Name of the laboratory**

**Comments:**

**It does not participate in any External Pre-analytical Quality Assurance Program**

**What is your main reason for not participating in an external pre-analytical quality assurance programs?**

1. High cost
2. I don't deem it necessary to monitor the pre-analytical phase
3. Results are not useful
4. I don't know how to interpret the results obtained
5. Data collection difficulty
6. I was not aware of the availability of this Program
7. I use indicators other than those of the Program
8. Other

**If your laboratory collects pre-analytical QI other than those in the Program, please, indicate which ones:**

**How do you collect the data required for calculating the QI?**

1. Through the Laboratory Information System
2. By manual counting on Excel (or similar)
3. By retrieving data from the Information System of the centre
4. Other

**What is your LIS supplier?**

**When you receive the results, does your laboratory review the indicators disaggregated by type of extraction centre? For example, if extractions are performed at different primary care centres and an indicator is elevated, do you review the results for that indicator in each of the centres?**

1. Yes
2. No

**Would it be useful to include Sigma calculations in the Program?**

1. Yes
2. No

**Would it be useful that the Program was extended to urgent samples?**

1. Yes, it would be useful to include indicators for urgent samples, along with routine samples in the same Program
2. Yes, a Pre-analytical program that included urgent samples separately from routine samples would be useful
3. No

**Would you be interested in participating in a Post-analytical Program?**

1. Yes
2. No

**What post-analytical indicators would be more useful for you?**

1. Number of reports returned out of the specified deadline/Total of reports returned
2. Turnaround time from receipt at the laboratory to the reporting of results for potassium in urgent samples (p90)
3. Turnaround time from receipt at the laboratory to the reporting of results for INR in urgent samples (p90)
4. Turnaround time from receipt at the laboratory to the reporting of results for the hemogram in urgent samples (p90)
5. Turnaround time from receipt at the laboratory to the communication of results for TnI in urgent samples (p90)
6. Percentage of critical results alerted/Total of critical alerts raised
7. Other

**In relation to your laboratory, please mark the number of requests received annually**

1. < 25,000
2. 25,000 - 300,000
3. 300,000

**In relation to your laboratory, please mark the type of requests received**

1. Routine ambulatory
2. Urgent ambulatory
3. Routine hospital
4. Urgent hospital

**What percentage of external samples does your laboratory receive? External samples are defined as samples not extracted at the centre.**

1. < 33%
2. 33 - 66%
3. 66%

**Name of the laboratory**

**Comments:**
